# Breaking the Link between Environmental Degradation and Oil Palm Expansion: A Method for Enabling Sustainable Oil Palm Expansion

**DOI:** 10.1371/journal.pone.0068610

**Published:** 2013-09-06

**Authors:** Hans Harmen Smit, Erik Meijaard, Carina van der Laan, Stephan Mantel, Arif Budiman, Pita Verweij

**Affiliations:** 1 Copernicus Institute of Sustainable Development, Utrecht University, Utrecht, The Netherlands; 2 REDD+, Energy and Agriculture Program (REAP), Netherlands Development Organization (SNV), Jakarta, Indonesia; 3 People and Nature Consulting International, Jakarta, Indonesia; 4 School of Biological Sciences, The University of Queensland, Brisbane, Australia; 5 Center for International Forestry Research (CIFOR), Bogor, Indonesia; 6 ISRIC World Soil Information, Wageningen, Netherlands; 7 Ecology and Environment, Padjadjaran University, Bandung, Indonesia; 8 World Wide Fund for Nature Indonesia, Jakarta, Indonesia; U. S. Salinity Lab, United States of America

## Abstract

Land degradation is a global concern. In tropical areas it primarily concerns the conversion of forest into non-forest lands and the associated losses of environmental services. Defining such degradation is not straightforward hampering effective reduction in degradation and use of already degraded lands for more productive purposes. To facilitate the processes of avoided degradation and land rehabilitation, we have developed a methodology in which we have used international environmental and social sustainability standards to determine the suitability of lands for sustainable agricultural expansion. The method was developed and tested in one of the frontiers of agricultural expansion, West Kalimantan province in Indonesia. The focus was on oil palm expansion, which is considered as a major driver for deforestation in tropical regions globally. The results suggest that substantial changes in current land-use planning are necessary for most new plantations to comply with international sustainability standards. Through visualizing options for sustainable expansion with our methodology, we demonstrate that the link between oil palm expansion and degradation can be broken. Application of the methodology with criteria and thresholds similar to ours could help the Indonesian government and the industry to achieve its pro-growth, pro-job, pro-poor and pro-environment development goals. For sustainable agricultural production, context specific guidance has to be developed in areas suitable for expansion. Our methodology can serve as a template for designing such commodity and country specific tools and deliver such guidance.

## Introduction

Land degradation is a growing global concern [1,2]. This process of adverse changes in the biophysical environment not only results in a decline in the productive potential of biomass, it also negatively impacts the provisioning of environmental services [3]. A recent study by the Food and Agriculture Organization of the United Nations (FAO) shows that globally 25% of the land is now highly degraded and 8% is moderately degraded due to soil erosion, water degradation and biodiversity loss. About 36% is stable or slightly degraded and 10% is ranked as ‘improving’ [4]. With on-going human population growth, the area of degraded land is likely to expand significantly [5] making mitigation of land degradation a global environmental priority.

Land degradation is often thought of in relation to desertification and in that context defined as an implicit loss of primary productivity in the terrestrial bio-productive system [6]. The interpretation of such losses in biological productivity and associated economic benefits depends however on the perspective, e.g. socio-economic or ecological. Someone’s degraded land can be someone else’s garden [7]. For example converting woodland into cropland can cause a decrease in biological productivity and the ability to produce products such as firewood, but meanwhile this conversion can increase the economic benefit of food production [6]. Defining what land degradation means for different stakeholders is therefore important, while improved quantitative and qualitative guidance regarding its identification would also be an important step towards improved land management [8–10]. Such guidance requires a holistic landscape approach with objective metrics that take into consideration different perceptions of degradation and how it impacts people, their livelihoods, and their environment [8,11].

One particular type of land degradation takes place in forest landscapes where agricultural expansion drives forest conversion while part of the cleared land is not or only temporarily utilized for agriculture [12–15]. In the face of increasing global scarcity of arable land an often proposed solution to reduce forest conversion is to focus agricultural expansion and intensification on land that has already been degraded instead of opening up lands still covered in natural vegetation [8–10,16,17]. This strategy has been suggested in the context of agricultural expansion in general [8], as well as for specific commodities such as soy [9], rubber [18,19], palm oil [15,16], and bio-energy feedstock in particular [20–22]. Tropical forest areas are one of the last remaining biomes where large tracts of land suitable for agriculture are still available [23]. The need for a robust methodology of spatially delinking increased production by diverting agricultural expansion towards degraded areas is in particular important in these biomes because of their high conservation values [9,16,24].

In this study we have developed a methodology for the identification of degraded lands for sustainable agricultural expansion. To this end, we have selected relevant initiatives promoting sustainable agricultural development. Subsequently we translated the criteria or legislative requirements into spatially explicit and regionally relevant indicators to identify and map the risk for negative impacts of agricultural expansion from a social and environmental perspective according to these sustainability criteria. The methodology developed in this study can support: 1) nongovernmental organizations seeking to identify priority areas for conservation; 2) the private sector for identifying future agricultural expansion in areas that comply with sustainability criteria; 3) governments at both local and national levels to align their land-use planning for agricultural expansion with their commitments towards social acceptability and environmental sustainability; 4) scientists and land use planners to identify alternatives for agricultural expansion in areas with conservation value; and 5) global policy makers with regard to planning for biofuels and carbon sequestration.

Although we have designed the methodology in such a way that it is applicable to a broad range of commodities, we have developed the methodology specifically for oil palm ( *Elaeis* *guineensis* ), one of the most rapidly increasing crops in the world. Focus on this particular crop is important because oil palm grows best in the climatic zones harboring moist tropical rainforests [7,23], which are earth’s most biologically rich and endangered terrestrial ecosystems [25,26]. Oil palm expansion into forested areas can therefore lead to high biodiversity loss, especially at the local scale [7,13,23]. The crop has expanded mostly in Asia, but is considered to become a major driver of deforestation in tropical Africa [27] and Latin America [10,16,28] as well. At broader geographic scales and considering the growing global demand for vegetable oil, it remains unclear to what extent the high productivity potential of oil palm reduces pressure on land that would otherwise have been targeted by other oil crops [23]. What is clear though is that, considering the sustainable development targets of the sector, as well as broader sustainability targets of the main producing countries, appropriate land use planning is essential.

In developing our methodology to identify ‘degraded areas’ suitable for oil palm expansion, Indonesia was selected to test the method due to the urgent need for such for tools supporting spatial planning that avoid further deforestation [11,24,29,30].

Additionally, most palm oil is produced in Indonesia, which is equal to 46% of the total global production, on 6.2 million ha of land [31]. Between 1990 and 2005, 1.7–3.0 million ha of forest were cleared in Indonesia for the expansion of oil palm [6], making oil palm expansion one of the major drivers of deforestation. In the same period, FAO data indicate that Indonesia lost about 20.7 million ha of forest [6,23], which suggests that between 8 and 14% of Indonesia’s forest loss in that period could be attributed to oil palm plantation development. Indonesia is also one of the most biodiversity rich areas in the world [32], and because oil palm plantations harbour only 15% of the biodiversity of natural forests [33], oil palm expansion is considered an important driver of biodiversity loss and greenhouse gas emissions in Indonesia [34–36]. This poses a dilemma to Indonesia when attempting to meet the targets of *agri*-industrial expansion as well as environmental sustainability, biodiversity protection and poverty alleviation (e.g. Millennium Development Goals and targets of the Convention of Biological Diversity). Indonesia has to determine how it can reconcile these different goals.

Oil palm expansion on deforested or degraded land can be economically feasible [37] and is considered a sustainable option for Indonesia to meet the multiple objectives of socially and environmentally sustainable economic growth [11,38]. Identifying ‘degraded lands’ has, however, been a challenge as a result of a wide range of existing definitions [39,40], especially in the Indonesian context, where several definitions on degraded land exist in legislation [37]. The confusion in the discussions on what constitutes ‘degraded land’ has led to several methods for identification that only partially overlap. While such discussions continue, practical guidance on identifying areas that comply with the relevant sustainability standards has not received much attention. To move away from discussions on what constitutes ‘degraded land’ we propose a more pragmatic approach, by making the requirements that are relevant in the context of oil palm spatially explicit.

For the development of the methodology, we have translated the criteria from the sustainability initiatives the Roundtable on Sustainable Palm Oil (RSPO), the Roundtable on Sustainable Biofuels (RSB) and the legislative requirements in the Renewable Energy Sources Directive (RES-D) into spatially explicit and regionally relevant indicators. Our work builds on earlier attempts to translate oil palm sustainability and suitability criteria into spatially explicit indicators in four pilot studies in Indonesia [41–44].

By applying the methodology to West Kalimantan, Borneo, we demonstrate that it can contribute to breaking the link between oil palm expansion and environmental degradation, by 1) selecting priority areas for sustainable expansion according to leading sustainability standards, 2) visualizing the potential impacts of applying these sustainability standards, 3) providing guidance on trade-offs between (agricultural) economic development and sustainable land-use planning. These considerations are important to global policy development in the sustainability context of bio energy feedstock production and agricultural expansion in general.

## Methods

### Ethics

No specific permits were required for the described field studies.

### Methodological background

Many studies have attempted to spatially identify degraded land, although not necessarily in relation to agricultural expansion, including the Global Assessment of Soil Degradation (GLASOD) [45], the Assessment of the Status of human-induced soil degradation in South and South-East Asia (ASSOD) [46], and the Global Soil Degradation Assessment (GLADA) [5]. These efforts coincided with the emergence of the idea to utilize degraded land as a land-use planning strategy to prevent further environmental degradation [13,38]. The extent to which the degraded land identification studies of GLASSOD, ASSOD and GLADA can support land-use planning at the local level is, however, limited due to their low spatial resolution. In addition, the GLASOD and ASSOD studies focused on *soil* degradation, due to wind and water erosion, nutrient depletion and pollution, and are therefore of limited use to the present discussion on *land* degradation and deforestation as a consequence of agricultural expansion [47]. In GLADA, a different approach was used and degradation was assessed at the global scale in terms of changes in net productivity potential of biomass [5]. Although the objective is closer to our aims, the application of these results for our purpose is limited, not only because of their low spatial resolution, but also due to the lack of information on the current vegetation cover. Although not entirely meeting our meeting our objectives, the focus on degradation of vegetation cover instead of soil degradation seemed a promising approach. Such an approach was also used in a study by World Wide Fund for Nature (WWF), which focused on the drivers of degradation to identify degraded areas, and assessed the potential of degraded land for oil palm expansion from an agronomic point of view [37]. The WWF definition of degraded land was; ‘land where the native vegetation has been altered by anthropogenic activity resulting in a reduction in tree canopy cover, standing biomass or species diversity from which the system cannot recover unaided within a defined time period’. The study concluded that degradation of vegetation does not necessarily affect the land productivity potential. This means that means that high agricultural yields can be obtained on such lands, including from oil palm cultivation [37].

Although the WWF study was important for developing a definition useful for the identification of ‘degraded land’ and promoting the utilization of such areas as an economically viable strategy for sustainable oil palm expansion, it remains unclear how these areas could be identified in a spatially explicit way. Questions arise on the definitions of ‘native vegetation’, what the thresholds are for areas to be utilized in terms of biodiversity conservation and biomass accumulation and what timeframe should be considered for the recovery of the primary productivity of the ecosystem. Following a slightly different approach, Tyrie and Gunawan [48] designed a method to identify areas for sustainable forest management, areas for conservation and other uses by applying an approach that included degradation risk, capacity for forest regeneration, legal land status and biophysical land suitability. That method was applied at spatial levels ranging from individual concessions to the province, with a focus on forest management planning. In a later stage, the method was applied for all provinces of Kalimantan to assess potential land suitability for oil palm expansion. Because the study by Tyrie and Gunawan did not consider how their method aligned with international sustainability criteria, their method is difficult to use for companies and land uses planners. Our study adds to their insights by including more detailed environmental, social and biodiversity criteria and applying it to a sector that has fundamentally changed since.

For the development of the current methodology, we have built on the latter approach to define what was referred to as Responsible Cultivation Areas (RCA). We translated the criteria of three leading sustainability initiatives relevant in the context of sustainable oil palm expansion into spatially explicit indicators that can be used to verify compliance with those sustainability initiatives that were described in the introduction. We have visualized the risk for non-compliance with the sustainability criteria through a ‘risk indicator map’. To generate options for compliance with the sustainability criteria in existing land-use plans as well as for future plantation development, we have compared planned plantation concessions as well as legal options for expansion with the risk indicator map.

### Selection of criteria

In order to fulfill their requirements, sustainability standards such as RSPO, RSB, and RES-D require companies to conduct social and environmental impact assessments (see the RSPO Principles and Criteria (P&C) criterion 7.1, the RSB P&C (version 1) principle 2 and the RES-D [49] article 23). In addition to these general requirements, the initiatives specify areas considered unsuitable for agricultural expansion under these schemes. In the RSPO P&C, criterion 7.3 is formulated as follows: ‘New plantings since November 2005 have not replaced primary forest or any area required to maintain or enhance one or more High Conservation Values’ (*p. 40: RSPO, 2007*). An assessment of High Conservation Values (HCV) as defined by the HCV Toolkits [50,51] provides the guidelines for both the social and the environmental impact assessment for RSPO members and is mandatory for all new plantings. The criteria within HCV toolkits [50,51] have significant overlap with those set by the RSB and RES-D (Table 1). To develop a holistic methodology for spatially referenced indicators, we combined the most demanding criteria from the various sustainability initiatives. In general, the assessment criteria as defined by the HCV Toolkits and referred to by RSPO are the most demanding and are therefore used as the basis for the methodology. We complemented these criteria with RSB or RES-D criteria that are not covered in HCV toolkits. We included an additional social requirement of RSPO’s criterion 7.5, which requires Free, Prior, and Informed Consent of local stakeholders (FPIC) for the establishment of plantations on local peoples’ land [52], which is also defined as an important criterion in the P&C of the RSB (Principle 12a) [53]. Where the sustainability initiatives differ is in the aim of minimizing the emission of greenhouse gases, which is currently voluntary according to the RSPO P&C, but is represented by a major mandatory criterion in both the RSB and the RES-D [49,53]. The RSB requires a 50% emission reduction compared to its fossil fuel baseline and the RES-D requires a 35% reduction by 2017 (from January 2017 at least 50%). This requirement and the reduction targets are included in the methodology presented here.

**Table 1:** Overlap of requirements between RSPO Principles & Criteria [52], Renewable Energy Sources-Directive (Article 17) [49] and RSB Principles & Criteria [53]. For details on the criteria see File S2.

RSPO	RES-D	RSB
HCV 1.1	3b i	P1, P7a, P7c
HCV 1.2	3b ii	P7a
HCV 1.3	3b ii	P7a
HCV 1.4		P7a
HCV 2.1		P7a, P7c
HCV 2.2		P7a
HCV 2.3		P7a
HCV 3	3b ii	P7a
HCV 4.1		P7a, P7b, P9
HCV 4.2		P7a, P7b, P8
HCV 4.3		P7a, P7b
HCV 5		P5, P6, P12a
HCV 6		P12a
C7.5:FPIC		P2b, P12b

Although oil palm can be cultivated in an economically viable way in many different tropical settings through specific management interventions, its productivity often declines when cultivated under suboptimal conditions, for example in areas that are frequently flooded [54,55]. To maximize the practical application of the methodology, and to comply with all the sustainability criteria related to site selection, we also included indicators related to biophysical suitability of oil palm (see principle 3, 4 and 7) [52].

In developing a comprehensible and comprehensive methodology, we grouped related indicators to improve clarity and efficiency of its application as well as to demonstrate relations between indicators. To maximize the comprehensibility and uptake of this methodology, we have taken into account practical considerations by applying existing tools and data sources and retaining existing structures as much as possible.

Taking into account these considerations, we categorized the indicators needed to verify the criteria of the sustainability initiatives into six criteria for the methodology, which were in turn distributed under three principles: 1) biodiversity and ecosystem services; 2) provisioning services and land (use) rights; and, 3) biophysical and economic suitability (Table 2; see File S2 for details).

**Table 2:** Methodology of selected principles, criteria and indicators for identifying areas suitable for sustainable oil palm expansion based on sustainability criteria of RSPO, RSB and RES-D [49,52,53] (see for additional information S2).

Principle	Criteria	Indicator
1: Conservation values must be maintained or enhanced	1.1: Valuable biodiversity is protected or enhanced on a population, meta-population and ecosystem level	1.1.1: Formal protection and conservation areas (HCV 1.1)
		1.1.2: Distribution and habitats protected and endangered species (Red List, CITES) (HCV1.2 - HCV 1.3 - HCV 1.4)
		1.1.3: Endangered ecosystem intact landscapes, and large scale intact forest (HCV 2 & 3)
	1.2: Ecosystem services are maintained	1.2.1: Hydrological functions (HCV 4.1)
		1.2.2: Erosion risk (HCV 4.2)
		1.2.3: Buffer zones large scale fire (HCV 4.3)
		1.2.4: Carbon stocks
2: Human wellbeing is ensured and land (use) rights are respected	2.1: Community Use is respected	2.1.1: Provisioning services crucial for subsistence (HCV 5) or Cultural sites (HCV 6)
		2.1.2: Customary Land Rights
3: The area is biophysically suitable for oil palm cultivation	3.1: Suitable climate	3.1.1: Rainfall
	3.2: Suitable topography	3.2.1: Slope
		3.2.2: Elevation
	3.3: Suitable soil	3.3.1: Drainage
		3.3.2: Soil texture
		3.3.3: Soil depth
		3.3.4: Soil erosion risk
		3.3.5: Soil chemical properties

### Suitability classification and analysis

Following the development of the indicators (Table 2) we defined four risk classes (low risk, medium risk, high risk, unsuitable) and associated thresholds values. These threshold values (Table 3) follow the sustainability initiatives, literature and expert knowledge.

**Table 3:** Applied suitability classification (see File S2 for the list of sources used and analysis applied).

Indicator	Low risk	Medium risk	High risk	Very High risk
1.1.1: Formal protection and conservation areas (HCV 1.1)	No overlap with IUCN areas or conservation and protected areas and buffer zones.	Bufferzones1km		IUCN I–IV, IUCN V–VII, protected forest, Ramsar and national conservation areas
1.1.2: Distribution and habitats protected and endangered species (Redlist, CITES) (HCV1.2 - HCV 1.3 - HCV 1.4)	No overlap with distribution or habitats of protected and endangered species	Overlap with distribution of protected and endangered species	Overlap with habitat of protected and endangered species (HCV 1.3)*	Breeding grounds and nesting places, grazing/browsing for endangered species and temporal habitats for migratory species (HCV 1.3 & 1.4)*
1.1.3: Endangered ecosystem Intact landscapes, and large scale intact forest (HCV 2 & 3)	No overlap endangered ecosystems, important ecotone regions and large scale forest	Forest area >20000 ha plus buffer 3 km (HCV 2.1)	2 or more eco-tone regions (HCV 2.2) Endangered ecosystems	Rare ecosystems: Karst class 1, peat, fresh water swamp, mangrove, *hutankerangas*(heath forest), cloud rainforest: peat, mangrove, cloud rainforest (HCV 3)
1.2.1: Hydrological functions (HCV 4.1)	No overlap with water source/riparian zones, mangrove peat, karst or DAS super priority	DAS Super priority	Mangrove, peat, wetland and karst forest, cloud forest, *hutanpunggung*	Water sources (spring), riparian zones and buffer zones.
1.2.2: Erosion risk (HCV 4.2)	< 15 ton/ha/year	15-60 ton/ha/year	60-180 ton/ha/year	> 180 ton/ha/year
1.2.3: Buffer zones large scale fire (HCV 4.3)	No overlap with barriers for the spread of large scale fire, the area recently burned more than once in the last 10 years*		Overlap with barriers for the spread of large scale fire, but (partly) burned in the last 10 years*	Area contains barriers for large-scale fire i.e. large forest blocks or peat swap areas and not burned during the last 10 years*
1.2.4: Carbon stocks	Carbon stock. 0-60 ton/ha	Carbon stock 60-70 ton/ha	Carbon stock. 70-80 ton/ha*	Carbon stock >80 ton/ha
2.1.1: Provisioning services crucial for subsistence (HCV 5) or Cultural sites (HCV 6)*	Areas providing for <10% for subsistence *	Areas providing >10% < 25% for subsistence *	Areas providing >25% <50% for subsistence, or containing cultural sites *	Areas providing >50% for subsistence, or containing cultural sites *
2.2.2: Customary Land Rights*	No overlap with land rights *	Idle land; community interested to change the use *	Idle land; *tanahpera*, community not interest to change the use *	Active use of land (*tembawang;simpung*; *limbo*;*tana*'*jaka*; *tana' ulen*; *gupung*; community protected forest)*
3.1.1: Rainfall	1750-5000 mm	1500-1750 mm	1250-1500 mm	< 1250 mm; > 5000 mm
3.2.1: Slope	< 8%	8-15%	15-30%	> 30% (> 12°)
3.2.2: Elevation	< 200 m	200-500	500-1000 m	> 1000 m
3.3.1: Drainage	Well to moderately well	imperfect	Extreme; poor	Excessive; very poor; stagnant
3.3.2: Soil texture	Silt loam; sandy clay loam;silty clay loam; clay loam	Clay; silty clay, sandy loam; loam	Sandy clay; silt; loamy sand	Heavy clay; sand
3.3.3: Soil depth	> 100 cm	75-100 cm	50-75 cm	< 50 cm
3.3.4: Soil erosion risk	< 15 ton/ha/year	15-59 ton/ha/year	60-179 ton/ha/year	> 180 ton/ha/year
3.3.5: Soil chemical properties#	Well drained and deep mineral soils, such as Nitisols, Alfisols	Weathered and deeply developed mineral soils; Tropudults (Ultisol),	Shallow and infertile mineral soils, e.g. LithicDystrudepts (Inceptisol)	Infertile sands (Tropopsamment, Placaquods), soils with acid-sulphate potential (Sulfihemist, Sulfaquept, Sulfaquent)

* no data was found.

# Soils were evaluated for the following properties: Cation Exchange Capacity, Base Saturation%, Soil Nitrogen, Available Phosphorus, Exchangeable Potassium, Anion Fixation, Mineral Reserve, Soil Reaction, Aluminum Toxicity, Salinity, Acid Sulphate Potential (here only some examples of soils and their indicative limitations are mentioned, with examples of soil taxonomic (sub) units).

### Spatial analysis

The spatial analysis used to develop the risk indicator map for West Kalimantan is based on data that have been verified and which were thus assumed as sufficiently accurate (see Table 4). Seven data layers were produced and subsequently superimposed in line with Liebig’s law of the minimum [56], where the highest risk class in the layers is overruling all other lower risk classes.

**Table 4:** Distribution of the suitability classification for each indicator in West Kalimantan (including distribution of oil palm concessions (C), provincial land-use plan (LUP) and the Risk Indicator Map (RIM)) in hectares.

Layer	Low risk	Medium risk	High risk	Very High Risk
1.1.1	10,264,750 (79%)	171,285 (1%)	0	2,542,545 (20%)
1.1.2	8,002,697 (62%)	0	4,975,900 (38%)	0
1.1.3	6,180,760 (47%)	1,531,902 (12%)	4,093,223 (31%)	1,411,301 (11%)
1.2.1	4,888,399 (37%)	7,866,970 (60%)	307,058 (2%)	156,690 (1%)
1.2.2	8,820,942 (67%)	3,181,400 (24%)	814,944 (6%)	401,832 (3%)
1.2.4	8,411,319 (64%)	0	2,738,594 (21%)	2,061,619 (16%)
3.1.1	7,369,502 (56%)	10,780 (0%)	594,496 (4%)	5,244,338 (40%)
R.I.M.	2,615,081 (20%)	0	1,256,493 (10%)	9,107,006 (70%)
R.I.M. & C.	916,000 (7%)	0	523,886 (4%)	2,054,963 (16%)
R.I.M. & L.U.P.	521,802 (4%)	0	180,936 (1%)	1,068,394 (8%)

To develop practical and spatially explicit recommendations on the potential for sustainable expansion in line with the sustainability criteria, we additionally identified the land that is being classified as low risk in existing concessions. To this end we superimposed the risk indicator map on the existing concessions (plantation service West Kalimantan, 2007) (see Table 4). We differentiated between concessions that were ‘active’, i.e. formally operational, and ‘inactive’, i.e. not formally operational, but in the process of license application. This distinction is useful, because we can give guidance for sustainable expansion based on the existing land-use plans. This method can also be used to inform the Indonesian Government, which is increasingly minimizing licensing applications as land speculation tools rather than for serious plantation development.

In addition to assessing existing plans for plantation development, the risk indicator map was also used to assess future options for oil palm expansion in line with the sustainability criteria. To come to such recommendations, the risk indicator map was superimposed onto the provincial land-use plan for West Kalimantan (2005), i.e. the *Rencana Tata Ruang Wilayah Propinsi* (RTRWP). In the provincial land-use plan roughly two land-use types were classified; land for forestry and land for other use. Indonesian law only allows oil palm concessions on land designated for ‘other use’ (i.e. *Areal Penggunaan Lain*). Although the designation of the land can in principle be changed between either categories, in practice it is mostly uni-directional, i.e., from forested land to conversion forest (i.e. *Hutan Produksi Yang Dapat Di Konversi*). To identify and map areas recommended for plantation development that had a low risk of violating the sustainability standards, we accounted for these practical limitations in land-use planning and considered four classes (listed from high to low potential, respectively): land for other use (*Areal Penggunaan Lain*), conversion forest (*Hutan Produksi Yang Dapat Di Konversi*), production forest (*Hutan Produksi*, *Hutan Produksi Terbatas*) and forest for protection purposes (*Hutan Lindung, Hutan Suaka Alam*and *Hutan Wisata*).

## Results

**Figure 1:**
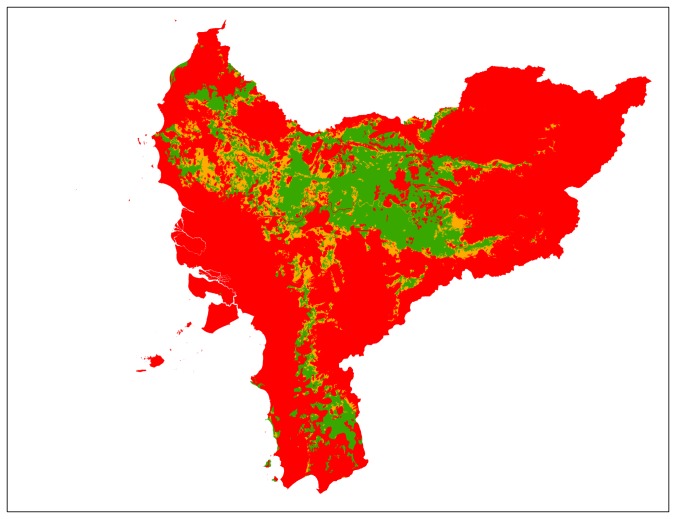
Risk Indicator Map, visualizing the risk of non-compliance with the sustainability standards. The distribution of land of each risk category (in hectares) is: Low risk (green), High risk (orange), Very High risk (red) * (see for additional information S1). *Since this is an assessment on the provincial level, this map is indicative and must be interpreted with caution regarding statements in the local context (see also discussion).

**Figure 2:**
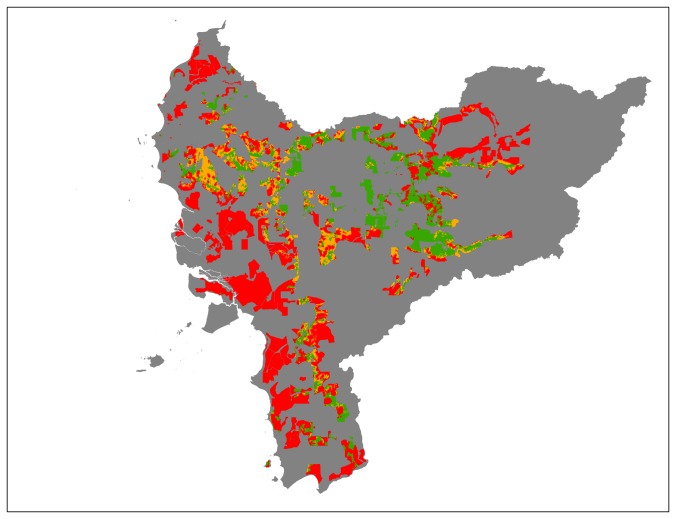
Inactive concessions superimposed on the risk indicator map. Low risk (green); High risk (orange); Very High risk (red).

**Figure 3:**
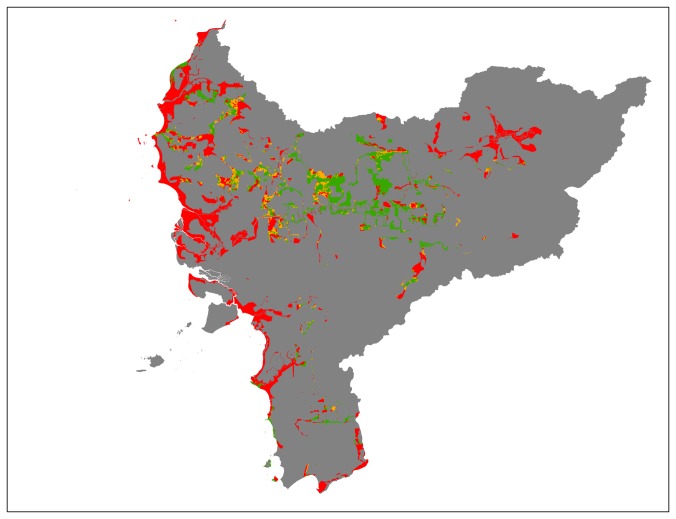
Land designated for ‘other use’ (*Areal Penggunaan Lain*) outside existing concessions. Low risk (green); High risk (orange); Very High risk (red).

According to our risk indicator map for West Kalimantan (Figure 1), the most limiting factors for sustainable oil palm expansion are HCV category 1.1 and biophysical suitability (see Table 4). By superimposing all layers in line with our suitability classification, we have found that 2,615,081 ha (20%) of the province has a low risk for non-compliance with the sustainability standards (see Figure 1 and Table 4). This is a substantial area when compared to the expansion rate of oil palm in this province (~40,000 ha per year). Even in existing inactive concessions we found that 914,853 ha have low risk of violating the sustainability standards. The results also show, however, that more than ~2,000,000 ha (almost 60% of the area in inactive concessions) is considered to have a very high risk to be non-compliant with the sustainability standards (see Figure 2 an Table 4). In assessing options for new concessions, we have found that 521,802 ha has a low risk of violating the sustainability criteria in land designated as ‘other use’ (*Areal Penggunaan Lain*) and land outside existing concessions (see Figure 3 and Table 4).

## Discussion

### Implications for land-use planning

In this study we have developed and tested an integrated spatially explicit methodology that supports the identification of candidate areas for agricultural expansion that have a low risk of displacing important social and environmental values. By using the criteria of established sustainability standards, specific guidance is provided for the target commodity. Applying this methodology for sustainable oil palm expansion in West Kalimantan resulted in a risk indicator map that visualizes the risk of violation of the selected sustainability standards. In addition, to move towards practical spatially explicit recommendations for future expansion in the context of land use planning, we superimposed the risk indicator map for West Kalimantan on the provincial land-use plans that constitute existing concessions and designated land-use.

In line with several existing studies related to degraded land identification as well as sustainable expansion areas for oil palm in Indonesia, we have found that ‘low risk’ areas for oil palm are a subset of the land cover categories grass and scrublands, often referred to as ‘degraded land’ [13,33,57]. In such areas the expansion of oil palm can have a positive effect on the environment by providing better protection of the soils from erosion [58] and even providing more biological value for forest species compared to grass and scrublands [33]. Replacement of such degraded vegetation types by commodities with high commercial value reduces the risk of large-scale fires, because potential financial losses from fire in plantations established on degraded soils would ensure that these are prevented [58]. Besides, oil palm expansion on these lands can also significantly increase carbon stocks for at least the lifetime of the planting cohort, which is being supported by earlier studies [13,58,59]. Considering the potential benefits, priority areas for expansion should be sought in grass and scrubland areas indicated as low risk on the risk indicator map.

The use of inactive concessions on low-risk land and using low-risk areas on lands under the responsibility of the Ministry of Forestry is currently constrained by the complicated procedures required to change land-use plans. The only class for which a status change is at present relatively easy is for conversion forest (*Hutan Produksi Yang Dapat Di Konversi*), but the amount of land designated as conversion forest and meanwhile considered as low risk is relatively small. There is an obvious need to develop simpler procedures to force inactive concessions to become active (or otherwise to cancel their permit), and to allow low-risk Ministry of Forestry lands to be used for oil palm plantation development. Presently such flexibility in land-use planning is lacking, making land-use optimization challenging.

Considering the expansion rate of oil palm plantations in West Kalimantan of about 40,000 ha annually [60], sufficient land classified as low risk should be available in existing inactive concessions (i.e. 916,853 ha) for at least several years. Allocating additional concessions in the short term seems therefore unnecessary. In case additional concessions are going to be allocated anyway, we strongly recommend to plan these concessions on land that has been classified as low risk in the areas designated as other use (521,802 ha). For example, planned expansion on forest areas in other provinces can be diverted to these areas. Only after that potential has been exhausted, forestry land, especially if heavily logged or damaged by fire, should be considered for expansion, and then we recommend to expand preferably on land identified as low risk in conversion forest *Hutan Produksi Yang Dapat Di Konversi* (227,245 ha).

### Strong points data and analysis

In developing indicators and criteria for this methodology, it was found that this provides pragmatic guidance on identification of areas for sustainable expansion for the commodity. This approach also allows comparisons between standards and regulations. For oil palm the systematic comparison of the criteria of the RSPO, RSB and RES-D sustainability standards, and their subsequent translation into spatially explicit indicators is useful for three purposes. Firstly, the identification of candidate areas for the sustainable expansion of oil palm that would be considered suitable under these schemes becomes more transparent, spatially explicit and regionally relevant. Secondly, the impact of the sustainability standards for biofuel feedstock cultivation is visualized. Thirdly, it provides specific recommendations for collection of field data. Since the indicators selected are based on leading sustainability initiatives, the results are useful for identification of land that can be utilized according to these sustainability initiatives and implementation of policy regulations. This is because 1) risks for violation of the sustainability initiatives can be mapped; 2) the implications of sustainability initiatives can be made spatially explicit; 3) conservation priorities can be identified through mapping areas with high conservation values under threat; and 4) land-use planners can be informed about options for minimizing risks regarding unsustainable development and maximizing trade-offs between economic development and conservation targets.

### Limitations and recommendations data and analysis

Due to inherent limitations of the quantitative and spatial data in the selected study area, our results should be interpreted by taking into account the following limitations. Potential errors exist in the data layer for vegetation cover as grassland/scrubland and land use for agriculture could not be distinguished reliably with the satellite data input used [61]. Some portion of the land that has been identified as ‘grassland’/scrubland can instead be part of a (shifting) cultivation system, and obviously oil palm expansion in such areas could cause conflict with current land-users. Grasslands are often hardly used by communities, due to the high mechanical and chemical input requirements. The scrublands, however, are potentially important for local food production [62]. Provisioning services of scrubland areas should therefore be investigated thoroughly, and dealt with through Free Prior Informed Consent (FPIC) procedures.

In addition, small patches of community forest areas or smallholder rubber could not be identified, due to the limited spectral resolution of 30m. Such areas can be important for biodiversity and for the provision of ecosystem services, especially water, carbon storage and food provisioning services. If part of a concession, the maintenance of such areas could and should be considered during plantation design. At the provincial level, such areas are easily overlooked during land-use planning. Hence the need for improved accessibility of legal land status maps and continued mapping and monitoring of land-use and cover. Any mapping and modelling based on our methodology should therefore be verified on the ground and address ecosystem and provisioning services on a case-to-case basis.

### Limitations of the methodology

The approach to use existing sustainability standards and regulations to provide guidance on the sustainable expansion of agriculture may not be sufficient for every commodity. Whether adequate spatial indicators can be extracted from existing regulations and sustainability standards depends strongly on how advanced the sector and the country is in terms of sustainability. For commodities and countries where such safeguards are not sufficiently implemented, lessons learned from the cultivation of crops with similar characteristics and contexts can be used to provide such guidance. Using the palm oil sector as a model is warranted due to the high level of its relevant sustainability criteria, and thus can provide useful input for sectors that are not as advanced in this respect.

Some indicators prove to be more important than others, depending on the importance of the criterion verified, as well as the availability and quality of verification data. In the development of the methodology, however, the exact relative importance of each indicator is not taken into account. In subsequent research, the indicators should be compared and assigned a ‘weight’. Assigning such weights can be done based on policy objectives and scientific insights, but should also take into account stakeholders views. In addition, to improve the practical value of the methodology, at least one additional indicator should be considered, i.e., proximity to infrastructure. For oil palm cultivation, the access to infrastructure is crucial since the fresh fruit bunches should be processed in oil palm refineries within 24 hours post-harvesting to retain good quality. Although new infrastructure can be developed, this may not be economically feasible depending on the size of the plantation. In addition, the development of new infrastructure in a previously closed area also leads to increased encroachment [63].

### Scientific and societal relevance

Sustainability initiatives are mostly the outcome of political negotiations rather than scientific debates. As a result, the criteria are often unclear and sometimes even ambiguous in their interpretation and implementation. By translating the criteria of sustainability initiatives into measurable spatially explicit indicators based on peer-reviewed literature, a scientific interpretation is provided and potential limitations of the initiatives are made explicit. This provides a new entry point for discussions on improving these initiatives, and a basis for developing more sustainable land use planning.

To increase global agricultural production, context specific guidance has to be developed in areas suitable for expansion [9]. Although our methodology is designed and tested for oil palm, the general framework and approach can serve as a template for designing such commodity and country specific tools. Even though intensification can mitigate the need for expansion, this strategy should be complemented by methods like the one developed in this article to ensure that increasing opportunity costs do not result in deforestation [9,10,64]. Our methodology is aiming to fill this gap. By making sustainability criteria spatially explicit, priority areas for sustainable development can be identified, as well as high risk areas that should be excluded from development. The impact of the sustainability schemes can be assessed and trade-offs between conservation and economic development targets can be identified. In addition, valuable strategic information is generated for stakeholders ranging from land-use planners, policy makers and corporate decision makers to civil society organizations. By testing this approach, we have found a variation of constraints regarding the lack of accurate spatial data, land speculation schemes and incoherent land-use planning. By implementing the methodology, we see several options for economic and sustainable development to co-exist in West Kalimantan and other tropical areas. Based on our findings, we conclude that in principle oil palm expansion in West Kalimantan does not necessarily have to be a driver of deforestation and degradation. Such envisioned sustainable development can, however, only be achieved through a collective effort of land-use planning agencies, civil society organisations, and the palm oil industry. Access to spatially explicit and accurate data is required: 1) to provide a basis for sustainable planning, 2) for successful development, enactment and enforcement of laws and regulations, and 3) for a fair and equal basis for negotiation between all stakeholders involved in land management and planning. A data-warehouse for spatial data is required to achieve this and data and tools for interpretation should be freely accessible. None of these proposed solutions are easy, but strong political will, collaboration between different government departments and levels of government (national, provincial, district), as well as a clear understanding of the social, economic, and environmental benefits should help break the link between environmental degradation and oil palm expansion.

## Supporting Information

File S1 Supporting online information for figure 1 (DOCX)

File S2 Supporting Online Information principles and criteria table 2 (DOCX)
