# Supplementary material for: Breaking the Link between Environmental Degradation and Oil Palm Expansion: A Method for Enabling Sustainable Oil Palm Expansion
Source: PLoS One. 2013 Sep 6;8(9):e68610. doi: 10.1371/journal.pone.0068610 (PMC3765141; doi:10.1371/journal.pone.0068610)
Supplement: File S1 — (DOCX) [file pone.0068610.s001.docx]

**S1: Supporting online information for figure 1**

***Data used and analysis applied in generating Risk Indicator Map***

- 1.1.1: Formal protection and conservation areas (HCV 1.1)
  1. Protected forest = *Spatial Planning unit Provincial Government West Kalimantan, 2005* Protected Forest (source: Spatial Planning unit Provincial Government, BAPPEDA , West Kalimantan).
  2. Forest status 2002 (source; Forestry service, 2010)
  3. Conservation Areas = Indonesian Conservation Areas 2007 (source: Ministry of Forestry/JICA 2010)
  4. IUCN I – IV = IUCN conservation areas categories I – IV (source: IUCN – UNEP, 2007).
  5. IUCN IV – VII = IUCN conservation areas categories IV – VII (source: IUCN – UNEP, 2007)
  6. RAMSAR = RAMSAR Areas (source: The Ramsar Convention on Wetlands of International Importance). Ramsar sites in 2004 (Ramsar Convention on Wetlands, 2006)
  7. Bufferzones = Conservation areas + 1 km
- 1.1.2: Distribution and habitats protected and endangered species (Red List of Threatened Species, CITES) (HCV1.2 -1.4)

1. Habitat endangered species (Orangutan): WCMC, 2004; Orangutan distribution map (based on regular surveys by WCMC, source www.unepwcmc.org). This data is used to assess the presence of orangutan habitats proximate to the concessions with Ian Singleton, 2004; Orangutan Population and Habitat Viability Assessment. This map is a contribution of the IUCN/SSC Conservation Breeding Specialist Group and areas with no forest cover (based on land cover West Kalimantan) classified as 2, and with forest cover classified as 3.
2. Land cover West Kalimantan (source: WWF, Indonesia ((1:50,000) -- Output 30 m)
3. (Temporal) habitats = EBA, endemic bird areas (source: Birdlife International, 2004)

- 1.1.3: Endangered ecosystem Intact landscapes, and large-scale intact forest (HCV 2&3)
  - Land system classification for Indonesia, (source: Ministry of Agriculture Indonesia). This data is used to assess the type of landscape present based on: parent material, hydro climate, landform, soil and vegetation (RePPProT, 1989, 1: 250,000)
  - Land cover West Kalimantan, 2009 (source: WWF Indonesia)
- 1.2.1: Hydrological functions (HCV 4.1)
  - Priority Water Catchment Areas, 1999 – 2009 (Source Ministry of Forestry). This data is used to identify watersheds. Priority Areas are watersheds classified by priority for conservation (the Ministry of Forestry defined criteria related to the status of the land in terms of erosion and land cover, water quality, water use, the context in the land-use plan [[32](#_ENREF_32)].
  - Rivers, 2008 (source: Interactive Atlas of Indonesia's Forests FOMAS)
  - Land cover West Kalimantan, 2009 (source: WWF Indonesia)
  - Asian Karst Data (University of Auckland, 2007); Asian Karst Data (source: School of Environment, The University of Auckland, http://www.sges.auckland.ac.nz/research/karst.shtml) This dataset is developed by Ford & Williams in 1989, the revision was published in 2007 was used here to identify unique karst ecosystems.
  - RePPProT, 1989
- 1.2.2: Erosion risk (HCV 4.2)
  - Erosion potential of West Kalimantan 2008 (Source: WWF, Indonesia).

1.2.4: Carbon stocks

- - Land cover West Kalimantan, 2009 (source: WWF Indonesia)
  - West Kalimantan Peatland Areas Wetlands International 2002; West Kalimantan Peat Land Areas (source: Wetlands International, 2002). This data is used to identify peat swamp forest.
- 3 Agricultural suitability:
  - mapping by ISRIC high and low input scenarios (uses RePPPRot 1978 data as input for landform, land use and information on soils compiled from various sources,; uses National Master-plan for Forest Plantations 1994a for climate inputs)

Land status

- Forest status 2005 (source; Forestry service West Kalimantan)

Plantations 2007 (source: plantation service West Kalimantan)
