# Supplementary material for: Breaking the Link between Environmental Degradation and Oil Palm Expansion: A Method for Enabling Sustainable Oil Palm Expansion
Source: PLoS One. 2013 Sep 6;8(9):e68610. doi: 10.1371/journal.pone.0068610 (PMC3765141; doi:10.1371/journal.pone.0068610)
Supplement: File S2 — (DOCX) [file pone.0068610.s002.docx]

**S2: Supporting Online Information principles and criteria table 2**

***Principle 1: Biodiversity and ecosystem* services**

The HCV criteria were originally divided in three categories: criteria on biodiversity (1-3), criteria on environmental services (4) and social criteria (5 & 6) [[1](#_ENREF_1)]. Here we group the criteria on biodiversity and ecosystem services under one principle: ‘Conservation values must be maintained or enhanced’.

***Biodiversity***

To identify areas important for biodiversity conservation, the High Conservation Value Toolkit refers to the standards provided by the International Union for the Conservation of Nature (IUCN)[[1](#_ENREF_1)]. IUCN developed the Red List of Threatened Species to identify species threatened with extinction. One of the major contributions of Red List’s assessments is spatial data on species distribution**.** These data are used in designing and planning conservation areas ranging from local and regional to global scale [[2](#_ENREF_2),[3](#_ENREF_3)]. In line with the HCV Toolkit[[4](#_ENREF_4)], here the Red List and the associated species distribution data is used, and complimented with additional data sources where needed to meet the requirements of the sustainability criteria.

***Ecosystem Services***

It has been demonstrated that the spatial distribution of biodiversity and ecosystem services do not necessarily overlap[[5](#_ENREF_5),[6](#_ENREF_6)]. Identifying areas crucial for maintaining ecosystem services is thus important to include in the analysis. The sustainability initiatives all recognize the importance of ecosystem services; however, the ecosystem services that are considered do not entirely overlap. The HCV Toolkit specifically focuses on ecosystem services that are important for preventing natural disasters like flooding, landslides and large-scale fire [[1](#_ENREF_1)]. Under the RSB P&C, these ecosystem services are also recognized, but in addition carbon sequestration and storage is included[[7](#_ENREF_7)]. In the RES-D, carbon sequestration and storage is also a key factor in determining the suitability of an area[[8](#_ENREF_8)]. Therefore, in addition to the ecosystem services as included in the HCV assessment, we include carbon sequestration and storage in the suitability analysis. The ecosystem services we consider are related to hydrology, erosion control, fire control, and carbon sequestration and storage.

***Principle 2: Provisioning services and land (use) rights***

Apart from the environmental impact of land-use changes associated with plantation development, the socio-economic impact on local communities must also be assessed (RSPO principle 6 and 7; RSB Principle 5, 6 and 9)[[7](#_ENREF_7),[9](#_ENREF_9)]. For compliance with RSB, areas important for food and water provision cannot be converted (principle 6 and 9 respectively)[[7](#_ENREF_7)]. For RSPO members, conversion of an area that qualifies as HCV 5 (important for providing basic needs) or HCV 6 (important for cultural identity), is not allowed after November 2005 [[9](#_ENREF_9)]. Both RSPO and RSB members are also required to respect existing land-use rights (see RSPO Principle 2 and 7, RSB Principle 12 and in addition the UN declaration on the Rights of Indigenous Peoples 2007)[[7](#_ENREF_7),[9](#_ENREF_9),[10](#_ENREF_10)].

***Provisioning services (HCV 5)***

In the Indonesian context, land-use and land-use rights are strongly related, mostly due to the system of ‘Adat’, where land-use can justify claims of ownership [[11](#_ENREF_11)]. In general, ‘*Adat’* rights are poorly documented, and have to be assessed based on interviews and land-use assessments [[12](#_ENREF_12)]. Since community land use and land-use rights are strongly connected, it was found useful to combine the analysis on both issues in one assessment. The approach as outlined in HCV 5 and 6, was found to be a useful guideline in verifying both the RSPO as RSB criteria in principle. In the context of HCV, however, the assessment refers only to natural areas [[4](#_ENREF_4)]. We propose to extend the scope of the analysis on HCV 5 and 6, and include areas that are not considered ‘natural’, to cover all the requirements from the sustainability initiatives on both community land use and land-use rights. As stated in the Indonesian HCV toolkit:

*‘An area mapped as community agricultural land does not constitute an HCV 5 area as defined in the Toolkit, but it is no less important for meeting basic needs than areas identified as HCV 5 and must also be accommodated in spatial planning by the MU.’* (p 106. [[4](#_ENREF_4)])

Areas that are important for provisioning services are important to consider in both RSPO (principle 2, 5 and 6)[[9](#_ENREF_9)] as well as the RSB (principle 5 and 6)[[7](#_ENREF_7)]. Based on these requirements, land-use and land-use rights should thus not only be considered for forest areas. The second principle is therefore broader than just HCV 5 and 6 and includes land-use (rights) outside forest areas; **‘**Human wellbeing is ensured and land (use) rights are respected’.

***Cultural values (HCV 6)***

Indigenous communities often have sites in the proximity of the village that they consider ‘sacred’, due to the presence of cemeteries and attributed religious properties [[13](#_ENREF_13)]. For such sites, local communities can also make legitimate claims, and are specifically addressed in the context of RSPO, and unsuitable for conversion based on HCV 6[[9](#_ENREF_9)].

***Principle 3: Biophysical suitability***

Based on existing studies [[14](#_ENREF_14),[15](#_ENREF_15)], it was found that the relevant biophysical constraints are related to climate, topography and soil.

In the identification of areas for sustainable expansion, such limitations have to be taken into account, not only for pragmatic reasons, but also to comply with the RSPO P&C. According to criterion 4.2 and 7.2 of RSPO, proper use of topographical and soil information in order to ensure sustained yield is achieved [[9](#_ENREF_9)].

**References**

1. Jennings S, Nussbaum R, Judd N, Evans T (2003) The high conservation value forest toolkit. ProForest. Oxford (UK).

2. Rodrigues ASL, Pilgrim JD, Lamoreux JF, Hoffmann M, Brooks TM (2006) The value of the IUCN Red List for conservation. Trends in Ecology & Evolution 21: 71-76.

3. Brito D, Ambal RG, Brooks T, Silva ND, Foster M, et al. (2010) How similar are national red lists and the IUCN Red List? Biological Conservation 143: 1154-1158.

4. Indonesian, Resource Institute DC (2008) Toolkit for Identification of High Conservation Values in Indonesia.

5. Chan KMA, Shaw MR, Cameron DR, Underwood EC, Daily GC (2006) Conservation planning for ecosystem services. PLoS Biology 4: e379.

6. Benayas JMR, Newton AC, Diaz A, Bullock JM (2009) Enhancement of biodiversity and ecosystem services by ecological restoration: a meta-analysis. Science 325: 1121.

7. RSB (2010) Roundtable Sustainable Biofuels Principles & Criteria for Sustainable Biofuel Production. Roundtable on Sustainable Biofuels.

8. EU (2009) Directive 2009/28/EC of the European Parliament and of the Council of 23 April 2009 on the promotion of the use of energy from renewable sources and amending and subsequently repealing Directives 2001/77/EC and 2003/30/EC. Official Journal of the European Union: 16-61.

9. RSPO (2007) Roundtable on Sustainable Palm Oil Principles and Criteria for Sustainable Palm Oil Production.

10. UN (2007) United Nations Declaration on the Rights of Indigenous Peoples. UN: Washington 12.

11. McWilliam A (2006) Historical reflections on customary land rights in Indonesia. The Asia Pacific Journal of Anthropology 7: 45-64.

12. Afiff S, Lowe C (2007) Claiming indigenous community: political discourse and natural resource rights in Indonesia. Alternatives: Global, Local, Political 32: 73.

13. Wadley RL, Colfer CJP (2004) Sacred forest, hunting, and conservation in West Kalimantan, Indonesia. Human Ecology 32: 313-338.

14. Corley RHV, Tinker P (2003) The oil palm. Oxford: Wiley-Blackwell. 562 p.

15. Fairhurst T, Härdter R (2003) Oil Palm: Management for large and sustainable yields. Singapore: Potash & Phosphate Inst. 382 p.
